# 663. Epidemiology and outcomes of recurrent *C. difficile* infection among hematopoietic cell transplant recipients

**DOI:** 10.1093/ofid/ofad500.726

**Published:** 2023-11-27

**Authors:** Eduardo Sanchez, Elizabeth M Krantz, Steven A Pergam, Catherine Liu, Frank P Tverdek, Zahra Escobar

**Affiliations:** UW. Fred Hutchinson Cancer Center, Seattle, Washington; Fred Hutch Cancer Center, Seattle, Washington; Fred Hutchinson Cancer Research Center; University of Washington, Seattle, WA; Fred Hutchinson Cancer Research Center, Seattle, Washington; Seattle Cancer Care Alliance, Seattle, Washington; University of Washington, Seattle, Washington

## Abstract

**Background:**

Recent updates in Clostridioides difficile infection (CDI) treatment guidelines among hematopoietic cell transplant (HCT) recipients recommend fidaxomicin as first line therapy as it reduces the risk of recurrent CDI. We aimed to characterize HCT recipients with CDI and describe their clinical outcomes, including incidence of recurrent CDI.

**Methods:**

This retrospective cohort study included patients ≥18 years old who underwent HCT at Fred Hutchinson Cancer Center from January 2012 to December 2021 and were diagnosed with CDI between day -7 and day +100 relative to HCT (Figure 1). Chart review was conducted to capture demographic, clinical and outcome data, including data on episodes of recurrent CDI. Recurrent CDI was defined as a new episode of symptoms consistent with CDI and a positive CDI test within 12 weeks after completing primary treatment. We computed the cumulative incidence of recurrent CDI, with treatment completion for the initial CDI as time zero and death before recurrent CDI as a competing risk.

**Figure:**
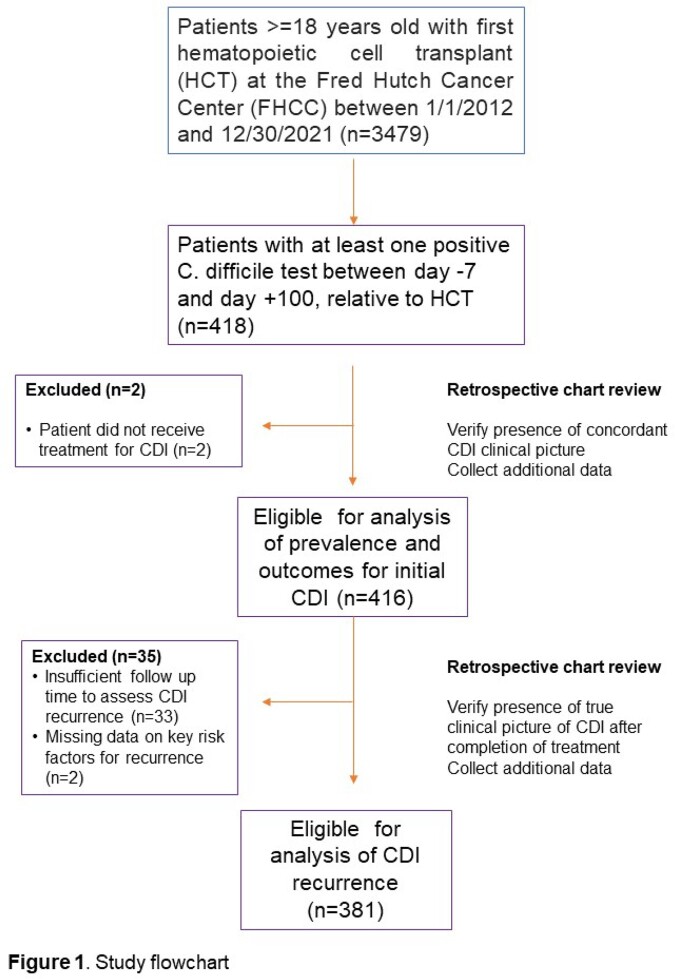

**Results:**

Of 3479 HCT recipients, 416 (12%) patients had CDI at a median of 5 days from HCT (Table 1). The most common underlying malignancies were acute leukemia and multiple myeloma; 248 (59%) patients received an allogeneic HCT. All but one patient were treated with metronidazole or oral vancomycin. Within 14 days of CDI, 38 (32%) patients of 119 diagnosed as outpatients required hospitalization and 3 (0.7%) of 410 not in the intensive care unit (ICU) required ICU level of care (Table 2); 10 (2%) died within 30 days of CDI diagnosis. Of 381 patients eligible for analysis of CDI recurrence, 30 patients had recurrent CDI at a median of 57 days (range, 1, 93) follow-up, for a cumulative incidence of 10% at 12 weeks (95% confidence interval, 7%, 14%, Figure 2). In the 14 days after CDI recurrence, 2 (10%) of 21 had a new hospital admission; none died within 30 days.

**Figure:**
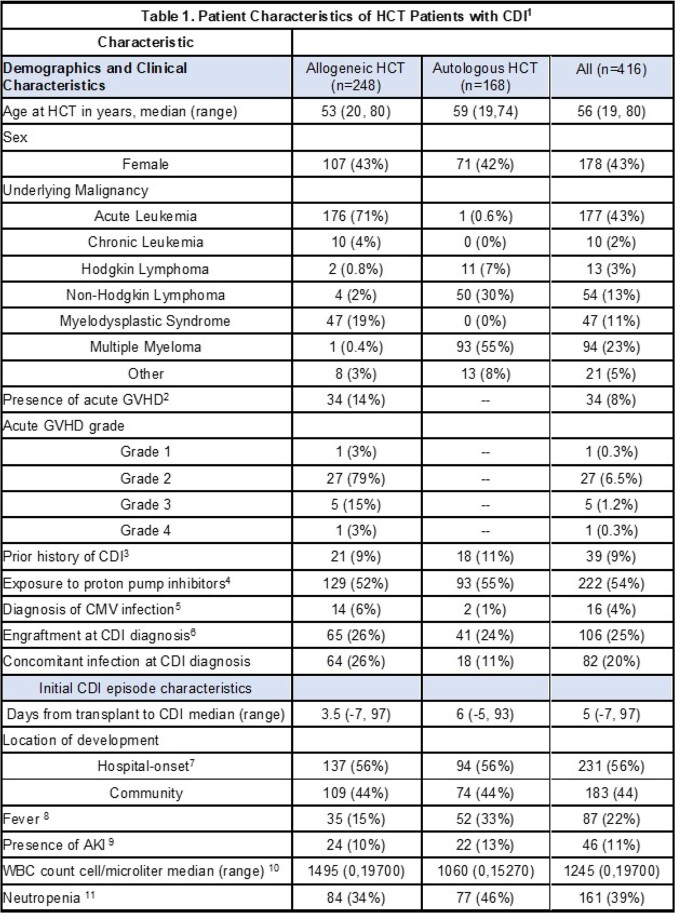

**Figure:**
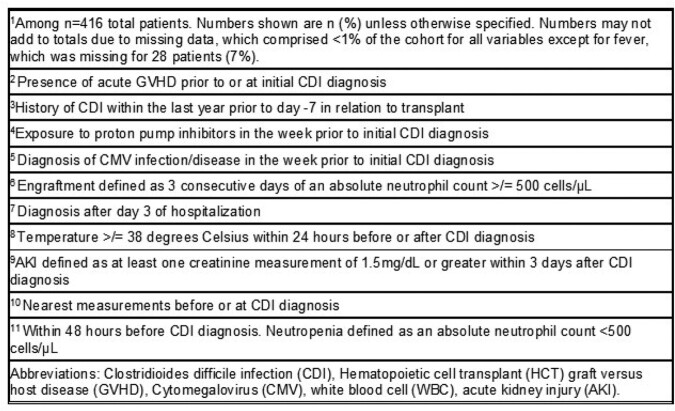

**Figure:**
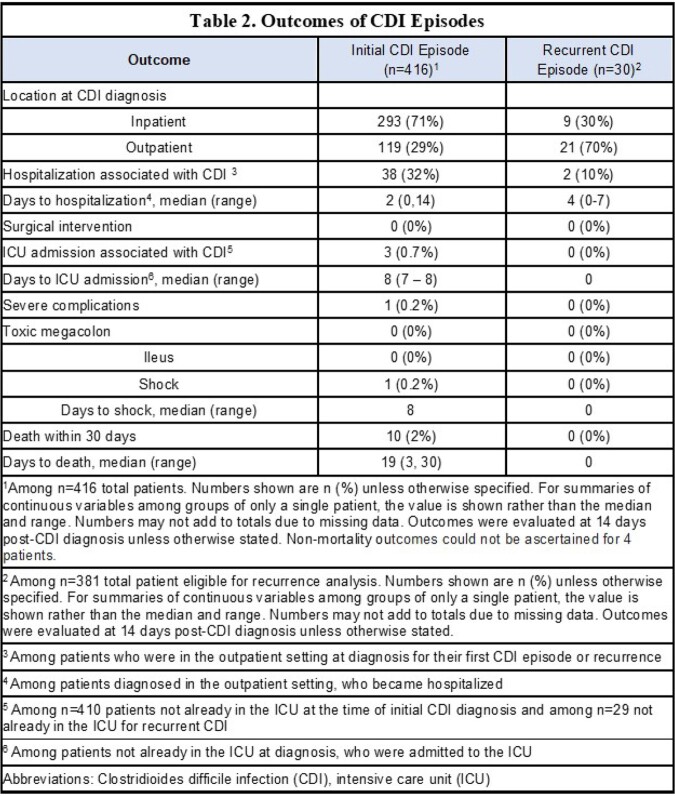

**Conclusion:**

The majority of HCT recipients in our cohort developed CDI early posttransplant; serious complications and deaths were uncommon. The incidence of recurrent CDI was low and may not warrant use of fidaxomicin for all initial CDI episodes in this population. Further work identifying HCT subgroups at higher risk of recurrent CDI may help to target fidaxomicin use to those most likely to benefit.

**Figure:**
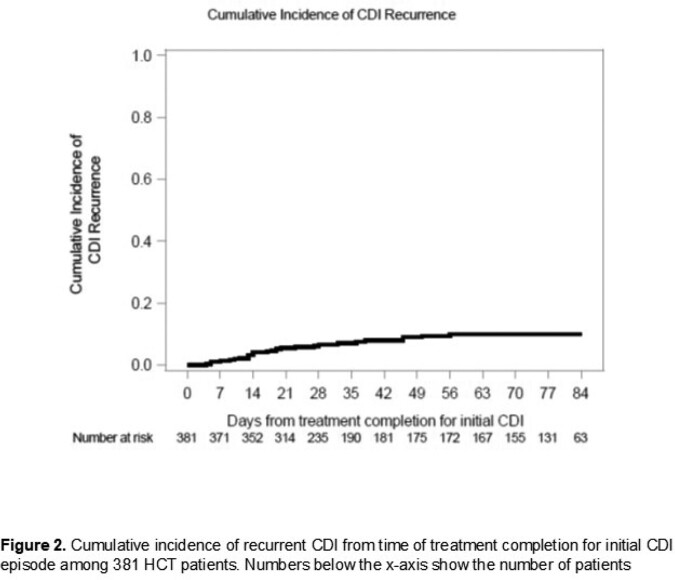

**Disclosures:**

**Steven A. Pergam, MD, MPH**, Cidara: Investigator in clinical trials|F2G: Investigator in clinical trials|Global Life Technologies: Grant/Research Support|Symbio: Investigator in clinical trials **Catherine Liu, MD**, Pfizer: Site Investigator|SNIPR BIOME: Advisor/Consultant

